# The Evaluation of Ovarian Function Recovery Following Treatment of Primary Ovarian Insufficiency: A Systematic Review

**DOI:** 10.3389/fendo.2022.855992

**Published:** 2022-04-28

**Authors:** Xiaojun Kuang, Yongzhe Tang, Hong Xu, Min Ji, Dongmei Lai

**Affiliations:** ^1^ The International Peace Maternity and Child Health Hospital, School of Medicine, Shanghai Jiao Tong University, Shanghai, China; ^2^ Shanghai Key Laboratory of Embryo Original Diseases, Shanghai, China; ^3^ School of Biomedical Engineering, Med-X Research Institute, Shanghai Jiao Tong University, Shanghai, China

**Keywords:** antral follicle count, follicle-stimulating hormone, ovarian function, anti-Müllerian hormone, primary ovarian insufficiency

## Abstract

**Background:**

Primary ovarian insufficiency (POI) is gaining awareness as its prevalence increases and its effect on patients is extremely negative. To date, several therapies have been designed to treat POI, but the conclusions are conflicting, in part, due to inconsistent evaluation methods. Thus, we explore a multi-index of ovarian function assessment methods to evaluate the recovery of ovarian function after various therapies in order to evaluate effectiveness in a more comprehensive manner.

**Aim:**

The purpose of this review is to assess the effectiveness of various therapies to recover ovarian function in patients with POI. The primary outcome measures were anti-Müllerian hormone (AMH) levels, follicle stimulating hormone (FSH) levels, and antral follicle count (AFC). The secondary outcomes included the change of mean ovarian volume, menstruation recovery, and pregnancy rate.

**Methods:**

Our systematic searching including PubMed, Web of Science, Cochrane, and Embase databases was conducted to find all human clinical trial articles published from January 2000 to April 2021 and related to POI treatment, including the keywords: POI, AFC, and hormones. All prospective and retrospective studies exploring ovarian function recovery that include AFC, AMH levels, and FSH levels evolution throughout treatment were included. All patients included in the studies met the POI criteria described by the European Society for Human Reproductive Embryology (ESHRE) guideline.

**Results:**

Six studies were selected based on the criteria: one randomized controlled trial and five observational studies. Among them, two studies focused on the intraovarian platelet-rich plasma (PRP) infusion treatment, two studies focused on dehydroepiandrosterone (DHEA) supplements, one study focused on hormone replacement therapy (HRT), and one study focused on autologous adipose-derived stromal cells (ADSCs) treatment. There was insufficient scientific evidence that any approach could help ovarian function recovery in patients with POI because the ovarian function markers in each study had inconsistent changes with 26 patients (6.2%) reporting spontaneous pregnancy.

**Conclusion:**

Serum AMH levels, FSH levels, and AFC are sensitive indicators and reflect the evolution of ovarian function. Large randomized controlled trials are necessary, and the data on ovarian function should be collected comprehensively to evaluate the effectiveness of a variety of treatments.

## Introduction

Primary ovarian insufficiency (POI) or premature ovarian insufficiency (POI) (1) is characterized by amenorrhea, elevated gonadotrophins, and decreased sex steroids, occurring in women under 40 years of age. POI is defined by the European Society of Human Reproduction and Embryology (ESHRE) guidelines as the presence of oligo/amenorrhea for at least 4 months and an elevated serum follicle stimulating hormone (FSH) level >25 IU/L on two occasions >4 weeks apart, with an onset before the age of 40 years (2). Over the past few decades, POI has become more common and has drawn more concern. The prevalence of POI occurs in about 1% of the population (2, 3). A recent meta-analysis showed that the prevalence of POI was as high as 3.7% (95% confidence interval: 3.1 - 4.3) (4). The consequences of POI range from the psychological damage associated with the diagnosis to the symptom burden (the loss of fertility), to the long-term consequences of decreased estrogen, including bone fragility and increased risk of life-long cardiovascular disease and neurocognitive disorders (5, 6). The causes of POI are variable, including genetic diseases (6, 7), microbial infections, viral infections (6), autoimmune diseases (8, 9), environmental toxins (10), and iatrogenic causes (2, 11). However, most cases of POI are idiopathic with unknown causes (2).

To date, there is still no effective therapy or intervention that can reliably improve residual ovarian function. Hormone replace therapy (HRT) is recommended for women with POI, both for symptom management and minimizing the burden from chronic disorders directly impacted by premature loss of estrogen (2, 12). For women who want to plan a family, assisted conception by *in-vitro* fertilization (IVF) using donor eggs could improve conception rates in women with POI. However, in many communities, this is not accepted either morally or socially (13). Furthermore, these strategies still could not restore ovarian function. Also, for patients not interested in future fertility, restoring ovarian function is an urgent need to improve their overall health condition. To date, most patients’ treatment studies have been focused on the achieved pregnancy (14, 15), but not the ovarian function recovery and the long-term improvement in ovarian function.

In women with POI, ovarian insufficiency can be semipermanent or intermittent and low ovarian function parameters are not always associated with the absence of follicles in the ovary (16). Indeed, Hubayter et al. had reported that 73% of patients with POI present antral follicles (17). Meanwhile, Kawamura et al. (18) found that up to 29.6% of patients with POI had follicular growth and developed preovulatory follicles within less than 6 months. However, studies have found that only 5–10% of women may become spontaneously pregnant after being diagnosed with POI (15).

Recently, antral follicle count (AFC), serum anti-Müllerian hormone (AMH) levels, and follicle stimulating hormone (FSH) levels have been used as valuable parameters of ovarian function (19) and also used as the marker for the prediction of POI (2, 20, 21). AMH expression is initiated in cuboidal granulosa cells of primary follicles as soon as primordial follicles develop, increases until the small antral follicles, and gradually diminish in subsequent stages of follicle development (20). AMH is only expressed in healthy follicles, but not in follicles that undergoing atresia (22). Also, AMH levels indirectly reflect ovarian function (23). Hansen et al. (24) compared forty-two healthy women’s ovarian primordial follicle number with AFC and hormone levels and found significant correlations between the primordial follicle count and AFC, AMH levels, and FSH levels. Rosen et al. (25) had investigated the relationship between AFC, AMH levels, and the age of Caucasian women (n=252) aged 25–45 years and found that AMH levels and AFC exhibited a significant correlation with age.

To date, there have been several clinical trials for patients with POI aimed at evaluating the recovery of ovarian function, such as intraovarian platelet-rich plasma (PRP) infusion and dehydroepiandrosterone (DHEA) supplements. Evaluating ovarian function by combining markers such as AFC, AMH levels, and FSH levels is necessary. Here, we review the literature in search of evidence for a real effect of such treatments to help recover ovarian function in women with POI.

## Materials and Methods

### Search Strategy

The systematic literature search was conducted in PubMed, Web of Science, Embase, and Cochrane databases covering the period January 2000 to July 2021. The systematic search used three keywords: POI (‘primary ovarian insufficiency’ or ‘premature ovarian insufficiency’ or POF (‘premature ovarian failure’) or ‘diminished ovarian reserve’ or ‘ovarian damage’), AFC (‘antral follicle count’), and hormone [AMH (‘Müllerian-inhibiting substance’ or ‘anti-Müllerian hormone’) or FSH (‘follicle stimulating hormone’)].

### Eligibility Criteria

For this Systematic research, only full-length articles in English with clinical observations in humans were included. This review incorporates all articles concerning ovarian function variation in patients with POI that meet the ESHRE criteria after various treatments, including randomized controlled trials (RCTs) and prospective clinical trials. We used AMH levels, FSH levels, and AFC as evaluation indicators of ovarian function, and all of them were required in the included articles. The principal summary measure was AMH levels, FSH levels, and AFC dynamics during and after treatment.

### Study Selection

**Figure 1:**
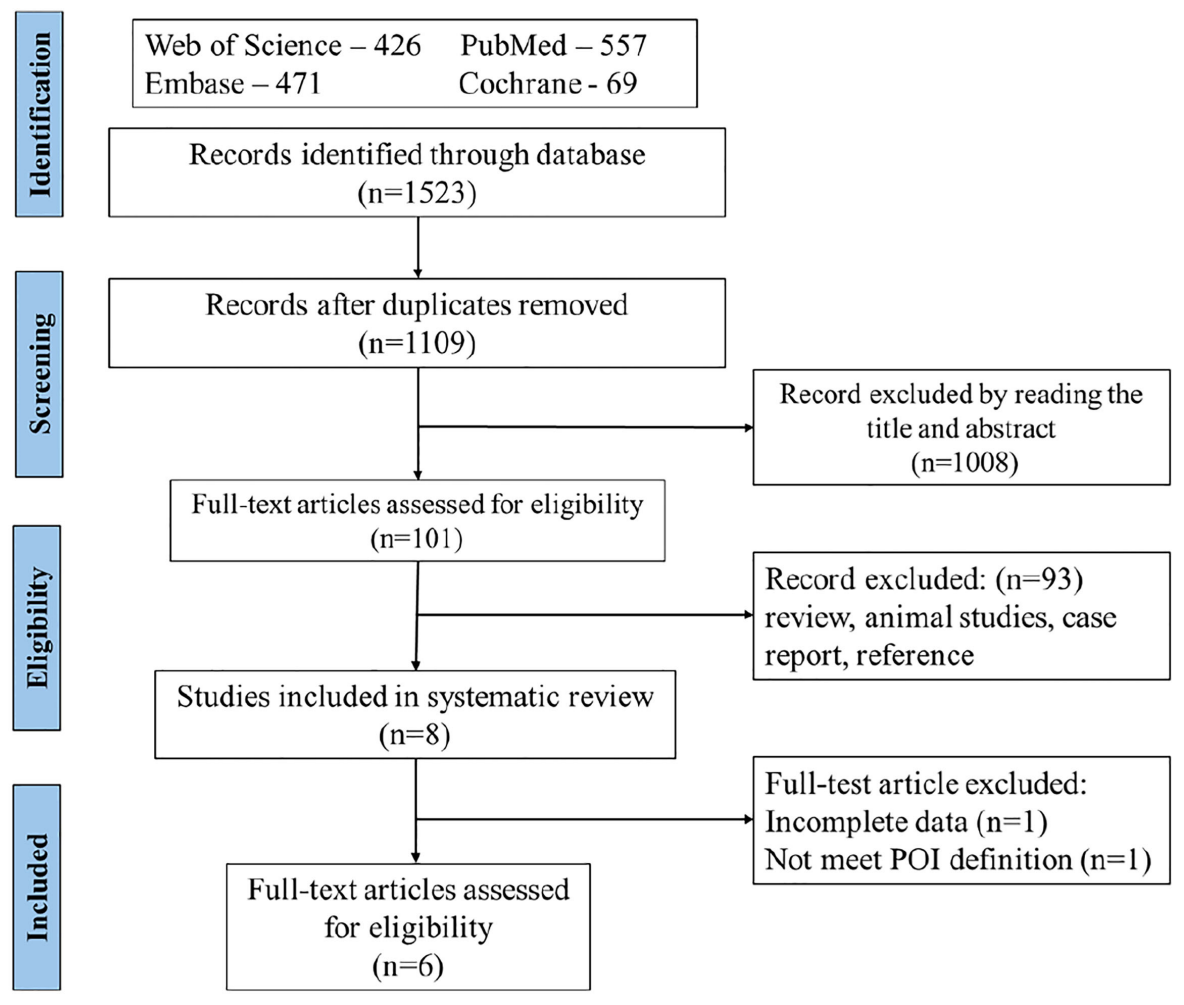
Flow diagram of identified studies.

Titles and abstracts of the identified studies were examined and irrelevant studies were deleted. The full text of potentially relevant studies was retrieved and evaluated, and if relevant, included in the study. A total of 1,109 studies met the search criterion after duplicates were removed. During the initial evaluation, 1,008 records were excluded based on title, abstracts, or both. During the second phase of the selection process of the remaining 101 studies, 93 records were excluded because they were irrelevant (review, animal studies, case report, and reference). In the final selection process, two studies were excluded because of incomplete data (n=1) and one did not meet the POI definition of ESHRE guideline (n=1). Finally, six studies were included in this review. The flow chart for the search results of our systematic review is described in **Figure 1**.

### Data Extraction

Data were collected using standard forms to document the characteristics of the study design, participants, intervention, comparisons, and main results. The data were extracted from the plots by WebPlotDigitizer (https://apps.automeris.io/wpd/index.zh_CN.html) if not given in the tables and texts.

### Outcomes

The primary outcomes were the changes in ovarian function markers, including AFC, AMH levels, and FSH levels following the treatments. Other markers, such as menses recovery, mean ovarian volume (MOV), and spontaneous pregnancy were secondary outcomes.

### Quality of Included Studies

The quality of the RCT evidence was judged to be moderate (26) according to the GRADE working group. The quality of evidence in observational studies was low.

## Results

### Description of Included Studies

Six eligible studies were selected in this review, including one randomized controlled trial and five prospective observational cohort studies. Four strategies for ovarian function recovery were used, including PRP treatment (16, 27), DHEA supplement (28, 29), HRT treatment (30), and intraovarian transplantation of autologous adipose-derived mesenchymal stromal cells treatments (31). The sample size of the individual studies ranged from 9 to 311, with a total of 421 patients. All patients met the definition of the ESHRE guideline (2). There were four patients with a history of chemotherapy (n=4), and three with chromosomal abnormalities (n=3) in one study (30), and the remaining patients had normal karyotypes and no history of radiation and chemotherapy. The main characteristics of the six studies are shown in **Tables 1**, **2**.

**Table 1:** Main characteristics of the six studies included in the review.

Reference and country	Design	Intervention	Number of patients	POI criteria	Characteristics of the population	Side effects
Yeung et al. (29); China	Randomized controlled study	DHEA or placebo 25 mg three times/day/16 weeks	22	1) Age < 40 years 2) FSH > 30 IU/L on two occasions of at least 6 weeks apart 3) Secondly amenorrhea	DHEA group (n=10) 1) Age (years): 35.9 ± 3.26 2) BMI: 21.4 ± 3.34 3) Duration of POI (months): 30 (2, 81) Placebo group (n=12) 1) Age (years): 33.4 ± 4.74 2) BMI: 21.1 ± 4.08 3) Duration of POI (months): 43 (6, 132)	No major adverse effects were reported
Wong et al. (28); China	Prospective observational study	DHEA 25 mg three times/day/12 months	31	1) Age < 40 years 2) FSH > 40 IU/L on two separate occasions 6 weeks apart 3) Amenorrhea or oligomenorrhoea	1) Age (years): 36 (31, 38) 2) BMI: 22.2 (20.2, 24.4) 3) Duration of POI (months): 20 (2, 180)	1) Acne: 5 patients (16.1%) 2) Transient polycythemia: 2 patients (6.5%)
Kasahara et al. (30); Japan	Prospective observational study	Equine estrogen 1.25 mg/day/10 days + (ethinylestradiol 0.05 mg and norgestrel 0.5 mg)/day/11 days, or equine estrogen 1.25 mg/day/21 days + chlormadinone acetate 2 tablets/day/11 days	19	1) Age < 40 years 2) FSH > 40 IU/L on at least two different tests 3) Amenorrhea more than 3 months	1) Age at onset of POI (years): 17.0 (15.0, 23.0) 2) Age at first observation period (years): 25.2 (23.4, 30.8) 3) Amenorrhea > 3 months	No side effects were reported
Cakiroglu et al. (16); Turkey	Prospective observational study	2 - 4 mL PRP were intraovarian injected into ovary	311	1) Age < 40 years 2) FSH > 25 IU/L on two occasions 4 weeks apart 3) Oligo/amenorrhea for at least 4 months	1) Age (years): 34.8 ± 4.3 2) Duration of infertility (years): 6.8 ± 4.9	No side effects were reported
Sfakianoudis et al. (27); Greece	Prospective observational study	4 mL PRP were intraovarian injected into ovary	30	1) Age < 40 years 2) FSH > 25 IU/L 3) Amenorrhea at least 4 months	Success group (n=18, 60%) 1) Age (years): 35.11 ± 1.57 2) Duration of amenorrhea (months): 10.06 ± 2.62 Failure group (n=12, 40%) 1) Age (years): 35.92 ± 1.93 2) Duration of amenorrhea (months): 10.17 ± 4.76	No side effects were reported
Mashayekhi et al. (31); Iran	Prospective observational study	Autologous ADCSs suspensions of 5 × 10^6^, 10 × 10^6^, or 15 × 10^6^ cells were transplanted into ovary	9	1) Age 20 – 39 years 2) FSH ≥ 25 IU/L on two occasions > 4 weeks apart 3) Amenorrhea at least 1 year	1) Amenorrhea > 1 year 2) Age (years): 32.3 ± 4.8 3) BMI: 24.7 ± 2.88 4) Secondary amenorrhea patients (n=8, 88.9%), primary amenorrhea 1 patients (n=1, 11.1%) 5) Diagnosed POI > 1 year	No side effects were reported

Results are given in mean ± SD, median (25%, 75%), median (rang), and number (rate, %).

**Table 2:** Ovarian function evolution of patients of the six studies included in the review.

Reference and country	Follow-up	FSH levels (IU/L)	AMH assay and AMH levels (ng/mL)	AFC	MOV (cm^3^)	Pregnancy	Menstruation
Yeung et al. (29); China	20 weeks	DHEA group Baseline: 82.8 (63.6, 142.7) After treatment: 70.0 (31.4, 113.8) Placebo group Baseline: 84.1 (73.5, 97.2) After treatment: 78.0 (62.3, 91.8)	Assay: AMH Gen II DHEA group Baseline: 0 (0) After treatment: 0 (0-0.11) Placebo group Baseline: 0 (0 - 0.13) After treatment: 0 (0 - 0.14)	DHEA group Baseline: 0 (0 - 2) After treatment: 2.0 (1 - 5) Placebo group Baseline: 0 (0 - 2) After treatment: 0 (0 - 3)	DHEA group Baseline: 0.75 (0.30, 0.9) After treatment: 1.90 (1.02, 3.02) Placebo group Baseline: 0.73 (0.60, 1.10) After treatment: 1.01 (0.80 - 1.55)	—	DHEA group Baseline: 0 After treatment: irregular menses [1 patients (11.1%)] Placebo group Baseline: 0 After treatment: irregular menses [3 patients (25%)]
Wong et al. (28); China	12 months	Baseline: 68.9 (57.4, 108.9) After treatment: 72.4 (54.3, 89.6)	Assay: Beckman Coulter Access 2 Baseline: 0.01 (0, 0.01) After treatment: 0.01 (0, 0.02)	Baseline: 0 (0, 1) After treatment: 0 (0, 1)	Baseline: 2.2 (1.4, 3.4) After treatment: 2.1 (1.3, 2.6)	—	Baseline: oligomenorrhoea [7 patients (22.6%)] After treatment: oligomenorrhoea [18 patients (58.1%)]
Kasahara et al. (30); Japan	—	AMH-negative group (n=14, 74%) Baseline: 46.4 (20.7, 31.2) After treatment: 33.0 (20.4, 49.0) AMH-positive group (n=5, 26%) Baseline: 66.2 (13.2, 78.5) After treatment: 10.0 (5.0 - 26.6)	Assay: picoAMH ELISA kit AMH-negative group Baseline: 0 After treatment: 0 AMH-positive group Baseline: 0 (0.0, 0.0) After treatment: 0.0077 (0.0046, 0.0223)	AMH-negative group Baseline: 0 After treatment: 2 patients (13.3%) had follicle growth AMH-positive group Baseline: 0 After treatment: 5 patients (100%) had follicle growth	—	—	—
Cakiroglu et al. (16); Turkey	6 weeks	Baseline: 41.6 ± 24.7 After treatment: 41.9 ± 24.7	Assay: not given Baseline: 0.13 ± 0.16 After treatment: 0.18 ± 0.18	Baseline: 0.5 ± 0.5 After treatment: 1.7 ± 1.4	—	Spontaneous pregnancy: 23 patients (7.4%)	—
Sfakianoudis et al. (27); Greece	3 months	Success group Baseline: 40.61 ± 6.05 After treatment: 20.67 ± 3.58 Failure group Baseline: 63.65 ± 6.41 After treatment: 59.40 ± 9.47	Assay: Roche kit Success group Baseline: 0.18 ± 0.04 After treatment: 0.75 ± 0.06 Failure group Baseline: 0.15 ± 0.04 After treatment: 0.30 ± 0.05	Success group Baseline: 0 After treatment: 2.33 ± 0.49 Failure group Baseline: 0 After treatment: 0	—	Success group: 3 patients (16.7%) Failure group: 0	Success group Baseline: 0 After treatment: 18 patients (100%) Failure group Baseline: 0 After treatment: 0
Mashayekhi et al. (31); Iran	12 months	Baseline: 66.9 ± 23.6 After treatment: 44 (mean)	Assay: not given Baseline: 0.13 ± 0.08 After treatment: 0.04 (mean)	Baseline: 0.6 ± 1.1 After treatment: 0.2 ± 0.7	Baseline: 2.8 ± 1.9 After treatment: 2.56 (mean)	—	Baseline: 0 After treatment: 4 patients (44.4%)

Results are given in mean ± SD, median (25%, 75%), median (rang), and number (rate, %).

### Ovarian Function Test Before Treatment

Participants in all 6 studies underwent ovarian function tests (included AFC, AMH levels, and FSH levels) before and after treatments. Before treatment, the mean (or median) AFC was similar across studies and ranged from 0 to 0.6. Mean (or median) serum AMH levels ranged from undetectable to 0.18 ng/mL. There was concern that the studies used four different AMH assays and in two studies the AMH assay methods were not reported. Before treatment, mean (or median) FSH levels varied significantly across the studies and ranged from 41.6 to 82.8 IU/L. Three studies had given the mean ovarian volume (MOV) data and the mean (or median) MOV before treatment varied across studies ranging from 0.75 to 2.8 cm^3^. Four studies had given the menstruation status before treatment and just 7 patients (22.6%) in one study reported oligomenorrhoea; all patients in other studies reported amenorrhoea.

### Randomized Controlled Trial

As presented in **Tables 1**, **2**, the RCT conducted by Yeung et al. (29) investigated the effect of DHEA on ovarian function in patients with POI. Twenty-two patients with unexplained POI were randomized and split into the DHEA group and placebo group. No significant changes were found in in AMH and FSH levels. AFC and ovarian volume of the DHEA group were significantly higher at the 12th week (2.00 range (1-5) vs. 1.00 range (0-2), *P* < 0.05) and the 20th week (median, 3.79 vs. 2.02 cm^3^, *P* < 0.05) after DHEA treatment compared to the placebo group. No significant difference in menses recovery was found between the two groups. This study found higher AFC in the DHEA group, while the other two markers (AMH levels and FSH levels) were not significantly changed.

### Prospective Observational Cohort Studies

A prospective observational study of 31 Chinese women with POI conducted by Wong et al. (28) had evaluated the long-term effect of DHEA in patients with POI. All patients had a normal karyotype and 19 (61.3%) patients had used HRT previously and HRT was withheld for at least two months before DHEA treatment, 24 (77.4%) patients were amenorrhoeic, and 7 (22.6%) patients were oligomenorrhoeic. There was no significant difference in FSH levels, AMH levels, and AFC after the DHEA treatment. This study concluded that there was no significant improvement in ovarian function by long-term DHEA treatment.

Nineteen patients with POI were described in the prospective observational study by Kasahara et al. (30). Of them, 5 patients lacked definitive causes for POI, 4 had histories of chemotherapy, 3 had chromosomal abnormalities, 7 were positive for thyroid autoantibodies, and all participants were treated with HRT. Patients with one or more detectable AMH level cycles were defined as AMH-positive group, otherwise defined as AMH-negative group. Five patients (26.3%, AMH-positive group) had increased AMH levels, while another 14 patients (73.7%, AMH-negative group) remained undetectable AMH during treatment. The concentration of serum FSH levels decreased significantly during HRT treatment in all patients and AFC had increased in 7 patients (50%) during HRT treatment. This study had found that 26.3% of patients had increased AMH levels and AFC and decreased FSH levels during the HRT treatment, while other patients had a significant decrease in FSH levels and unchanged AMH levels and AFC.

Recent studies showed that intraovarian injection of autologous PRP could enhance ovarian folliculogenesis (32). Thus, intraovarian injection of autologous PRP was hypothesized to improve ovarian function for patients with POI. In a prospective observational cohort study by Cakiroglu et al. (16), 311 patients with POI received transvaginal PRP treatment. All patients had a normal karyotype, had at least one ovary, and had more than a one-year history of infertility. After PRP treatment, AFC and AMH levels were significantly increased, while FSH levels did not change. There were 186 (59.8%) patients with no follicles detected before PRP treatment and this number decreased to 87 (30.0%) after treatment. There were 23 (7.4%) patients who had spontaneous pregnancy after PRP treatment; 11 patients delivered and 5 were ongoing between 24th to 35th weeks of gestation.

Another prospective observational cohort study of PRP treatment was conducted by Sfakianoudis et al. (27). After transvaginal PRP treatment, 18 (60%) of women with POI presented with menstrual recovery and reduced FSH levels that failed to be re-classified as POI. These women were regarded as a success group. The other 12 (40%) patients were regarded as failure group. All patients in the success group had a statistically significant improvement in AMH levels, FSH levels, and AFC after PRP treatment. However, there was no significant change in AMH levels, FSH levels, and AFC in other patients. Thus, this study found that about 60% of patients had increased ovarian function during the PRP treatment.

Finally, in the non-randomized clinical trial, Mashayekhi et al. (31) used stem cell therapy to restore ovarian function. Nine patients with POI were split into three groups (every group had 3 patients) randomly assigned to receive either 5 × 10^6^, 10 × 10^6^, or 15 × 10^6^ autologous adipose-derived stromal cells (ADSCs) transplanted into the ovary. All patients had a normal karyotype and none had FMR1 mutations. The concentration of FSH had decreased at the end of follow-up in all groups and there were no significant differences between the three groups. However, the AMH levels, AFC, and MOV were variable during the follow-up, and there were no significant changes after follow-up compared with the baseline, and there were no significant differences between the three groups. This trial found that the intraovarian embedding of ADCSs was associated with an inconsistent decline in FSH levels, but other ovarian function tests remained unchanged.

## Discussion

It is an unpleasant experience for a woman who has been diagnosed with POI, which means the loss of ovarian hormone production and infertility. HRT is recommended for POI treatment of symptoms of low oestrogen (2), which may prevent diseases of the cardiovascular system and bone loss, but need long-term treatment and have other health risks (33). Oocyte donation is an established option for patients with POI (6). However, this choice is not accepted in many regions. Therefore, the ovarian function restoration approach is important for women with POI.

FSH levels are one of the most common indicators for ovarian function and ovarian response after treatment. Indeed, FSH is an important indicator for POI diagnostic (2). However, FSH levels would change with the menses cycle (1). Moreover, the increased oestrogen levels, by adding exogenous oestrogen, would reduce FSH levels through the hypothalamic-pituitary-gonadal axis. Thus, the reduction in FSH levels during HRT therapy does not represent the recovery of ovarian function (34). Therefore, monitoring FSH concentration could only indicate ovarian function recovery for treatments without exogenous oestrogen.

Different from FSH levels, serum AMH levels are largely constant within and across several menstrual cycles and unaffected by additional exogenous hormones (35, 36). AMH is a promising indicator for ovarian function and is widely used as an ovarian function marker in patients after chemotherapy or radiotherapy (37). In addition, although these studies had not found the AMH levels as a useful marker during or after POI treatment and one of the possible reasons might be the absence of a more sensitive AMH assay. Most patients with POI had undetectable baseline AMH levels and a small range change of AMH levels might not be achieved with the existing assay (30). Therefore, a more sensitive AMH assay technique should be developed. Also, international standardization of the AMH determination is required for comparing the AMH result between different studies.

This review has several limitations, including the limited number of articles, small sample sizes, variable treatment methods, and variable quality of the included studies. Notwithstanding, this review systematically analysed the efficiency of POI treatment methods combined with various indicators of ovarian function markers, including AMH levels, FSH levels, and AFC, which provide guidance for future studies.

## Conclusion

POI may lead to long-term health complications due to the premature deprivation of ovarian sex hormones. With the rapid progress in the field of reproductive endocrinology, many potentially promising treatment modalities are already being explored. However, to date, there is still no effective method to restore ovarian function. It mainly lies in the lack of randomized clinical trials, large sample sizes, and unified indicators for ovarian function evaluation. It is recommended to combine multiple indexes to evaluate the effectiveness of future treatment modalities, such as AMH levels, FSH levels, and AFC. Among them, AMH is a sensitive ovarian function marker and an ultrasensitive AMH assay technique should be developed. In addition, large randomized controlled trials are necessary to comprehensively evaluate the improvement of ovarian function.

## Data Availability Statement

The original contributions presented in the study are included in the article/supplementary material. Further inquiries can be directed to the corresponding authors.

## Author Contributions

XK and YT: literature search, screening, data extraction, data analysis and manuscription draft. DL, MJ, and HX: manuscription modification. All authors reviewed the final version of the manuscript and approve it for publication.

## Funding

This work was funded by National Key Research and Development Program of China (No. 2018YFC1004800, 2018YFC1004802), the interdisciplinary program of Shanghai Jiao Tong University (YG2022ZD028 and YG2019QNA09), the National Natural Science Foundation of China (No. 81971334), and the Shanghai Municipal Council for Science and Technology (No. 20JC1412100).

## Conflict of Interest

The authors declare that the research was conducted in the absence of any commercial or financial relationships that could be construed as a potential conflict of interest.

## Publisher’s Note

All claims expressed in this article are solely those of the authors and do not necessarily represent those of their affiliated organizations, or those of the publisher, the editors and the reviewers. Any product that may be evaluated in this article, or claim that may be made by its manufacturer, is not guaranteed or endorsed by the publisher.

## Abbreviations

POI, primary ovarian insufficiency; AMH, anti-Müllerian hormone; FSH, follicle stimulating hormone; AFC, antral follicle count; PRP, platelet-rich plasma; DHEA, dehydroepiandrosterone; HRT, hormone replacement therapy; ADSCs, adipose-derived stromal cells; IVF, *in-vitro* fertilization; MOV, mean ovarian volume.
